# Effect and mechanism of downregulating the long-chain noncoding RNA TM4SF1-AS1 on the proliferation, apoptosis and invasion of gastric cancer cells

**DOI:** 10.1186/s12957-021-02334-y

**Published:** 2021-07-31

**Authors:** Chengzhi He, Wenjing Qi, Zhihui Wang

**Affiliations:** 1 Department of Gastrointestinal Surgery, Wuhan Central Hospital, Tongji Medical College, Huazhong University of Science and Technology, No. 26 Shengli Street, Jiang’an District, Wuhan, 430000 Hubei Province China; 2 Wuhan College of Arts and Sciences, Wuhan, 430345 Hubei Province China

**Keywords:** Gastric cancer, TM4SF1-AS1, Proliferation, Invasion, Apoptosis, EMT, TM4SF1, PI3K-AKT signalling pathway

## Abstract

**Background:**

To investigate long-chain noncoding TM4SF1-AS1 in gastric cancer (GC) tissues and cells.

**Methods:**

TM4SF1-AS1 in 40 GC tissues and adjacent tissues was detected and compared using real-time fluorescence quantitative PCR (qRT-PCR). TM4SF1-AS1 in MKN28 and SGC7901 GC cells was downregulated using small interfering RNA (shRNA). The cells were grouped into an interference group (shTM4SF1-AS1 group) and a control group (shControl group). MTT and Transwell tests were applied to determine the proliferation and invasion of the cells in both groups, and flow cytometry was performed to assess the apoptosis rate in the two groups. Western blotting was performed to determine changes in key proteins in cells during the epithelial-to-mesenchymal transition (EMT) and in the TM4SF1 and PI3K-AKT signalling pathways in response to the downregulation of TM4SF1-AS1.

**Results:**

The proliferation of MKN28 and SGC7901 in the shTM4SF1-AS1 group was significantly inhibited at 48 h and 72 h compared to that in the shControl group (all *P* < 0.05). In the shTM4SF1-AS1 group, the number of invaded MKN28 and SGC7901 cells was significantly lower than that in the shControl group (all *P* < 0.05). Apoptosis in the MKN28 and SGC7901 shTM4SF1-AS1 groups was significantly higher than that in the shControl group (all *P* < 0.05). Compared to those in the shControl group, levels of E-cadherin in EMT-related proteins were significantly elevated (*P* < 0.01), while levels of N-cadherin, Snail and Twist1 were significantly decreased (all *P* < 0.01). After silencing the expression of LncTM4SF1-AS1, the expression levels of TM4SF1 in the shTM4SF1-AS1 group were downregulated compared to those in the shControl group, and the p-PI3K and p-AKT proteins in the PI3K-AKT signalling pathway in the shTM4SF1-AS1 group were downregulated compared to those of the shControl group.

**Conclusions:**

TM4SF1-AS1 is upregulated in gastric cancer tissues and cells. Interfering with and downregulating its expression inhibit cancer cell proliferation, invasion and the EMT and promote apoptosis. The underlying mechanism for these effects is related to silencing the TM4SF1 and PI3K-AKT signalling pathways. TM4SF1-AS1 may be a potential therapeutic target for gastric cancer.

## Background

Gastric cancer (GC) is a malignancy of the human digestive tract common throughout the world. In 2012, 950,000 new cases were diagnosed, and approximately 723,000 deaths were reported, making this malignancy very life-threatening [1]. The common cause of GC is *Helicobacter pylori* infection, which accounts for more than half of the incidence of the disease. Other recognized risk factors are smoking, consumption of pickled vegetables and obesity [2]. GC patients are usually confirmed by pathological biopsy in endoscopy [3], and the primary treatment methods are surgery, radiotherapy, chemotherapy and targeted therapy [4]. Clinically, early GC can be cured. Unfortunately, GC does not have specific symptoms and is often diagnosed at an advanced stage. The prognosis of advanced GC is poor, and 5-year survival is less than 20% [5]. In-depth study of the molecular mechanism of GC is particularly important in the development of new and precise drugs for targeted treatment of GC and improving prognosis [6].

Long-chain noncoding RNAs (lncRNAs) are transcripts of greater than 200 nucleotides that have no obvious protein coding potential [7]. A large amount of evidence indicates that lncRNAs participate in the occurrence and progression of human malignancies by acting as oncogenes or tumour suppressor genes [8]. Transmembrane 4 superfamily 1-antisense 1 (TM4SF1-AS1) is the latest lncRNA molecule identified in non-small cell lung carcinoma (NSCLC). Its expression level is elevated in NSCLC carcinoma tissues and is correlated with lymph nodes and distant metastasis of NSCLC. It is also a molecular marker of poor prognosis in patients [9]. RNA sequencing of breast carcinoma revealed that expression levels of TM4SF1-AS1 in breast carcinoma tissues were higher than those in adjacent tissues [10]. However, the role and mechanism of TM4SF1-AS1 in GC are still unclear. This study explored TM4SF1-AS1 in GC tissues and its effects on the proliferation, apoptosis, invasion, metastasis and EMT process of gastric cancer cells. The mechanism by which TM4SF1-AS1 affects the biological process of gastric cancer tumours was also explored, providing a new theoretical basis for early diagnosis and targeted therapy of GC.

## Methods

### Main research materials

Selection of GC tissue specimens: 40 patients with GC undergoing radical gastrectomy between January 2019 and December 2019 in our hospital were included. Fresh GC tissues and adjacent tissues (at least 6 cm away from the carcinoma) were obtained during the operation and stored in liquid nitrogen for later use. All patients were diagnosed with GC for the first time and had not received any radiotherapy, chemotherapy, surgery or biotherapy before the surgery, with no tumours in other body regions. Patients who were not willing to participate in the research, pregnant women and patients with other parts of the tumour were excluded. Informed consent forms were obtained from all patients.

The MKN28, AGS, MGC803 and SGC7901 GC cell lines and the GES-1 normal gastric mucosa cell line were purchased from the cell bank of the Chinese Academy of Sciences. RNA extraction reagent, reverse transcription reagent and fluorescence quantitative PCR reagent of tissues and cells were obtained from Takara. Foetal bovine serum, trypsin, 1640 medium and Lipofectamine 2000 transfection reagent were purchased from Invitrogen, USA. The interference sequences of shRNA and interference control (shRNA-NC) of β-actin and TM4SF1-AS1 were designed and synthesized by Shanghai GenePharma Co., Ltd. Cell migration, invasion chambers and Matrigel (No. 354480) were purchased from BD. EMT-related primary antibodies against β-actin (c-47778), E-cadherin (sc-21791), N-cadherin (sc-8424), Snail (sc-271977) and Twist 1 (sc-81417) were purchased from Santa Cruz Biotechnology, Inc., USA. TM4SF1 primary antibody (PA5-21,119), PI3K-AKT signalling pathway protein PI3K (MA1-74,183) and AKT primary antibodies (44-609G), p-PI3K (PA5-104,853) and p-AKT primary antibodies (PA5-36,780) were purchased from Invitrogen, USA. Goat anti-rabbit/mouse secondary antibodies labelled with horseradish peroxidase (ab150113) were purchased from Abcam (USA). ECL developer (K22020) was purchased from Abbkine. An apoptosis detection kit (T6013) was purchased from Yuheng Biotechnology Company, Suzhou, China.

### Methods

#### Real-time fluorescence quantitative PCR (qRT-PCR) detection

Total RNA of GC tissues, paracarcinoma tissues, GC cell lines and transfected GC cell lines was extracted using TRIzol reagent and was then transcribed into complementary DNA (cDNA). The target gene was amplified by SYBR Green I reagent with cDNA as the template and β-actin as an internal reference, and the relative expression of the target gene was calculated using the 2^−ΔΔCt^ method.

TM4SF1-AS1 primer sequences: TM4SF1-AS1-F: **5′-3′** TGCAAGTCACTCTGATGCCG, TM4SF1-AS1-R: **5′-3′** AGCTCTGAGCAAACCATCCTC. β-actin primer sequences: β-actin-F: **5′-3′** GCACCCAGCACAATGAAGA, β-actin-R: **5′-3′** AATAAAGCCATGCCAATCTCA.

#### Cell culture, transfection and grouping

The GC cell lines MKN28, AGS, MGC803 and SGC7901 and the normal human gastric mucosa cell line GES-1 were routinely resuspended, placed in 1640 medium (containing 10% foetal bovine serum) and cultured in a 5% CO_2_ incubator at 37 °C. The expression levels of TM4SF1-AS1 in GC cell lines and normal gastric mucosa cells were detected, and MKN28 and SGC7901 cell lines were used for subsequent experiments. Cells in the logarithmic growth phase were inoculated in a 6-well plate at 5 × 10^5^ cells/well. After 24 h, they were transfected into TM4SF1-AS1 of the shTM4SF1-AS1 group and shControl group according to the transfection reagent instructions. The TM4SF1-AS1-shRNA sequence (5′-3′) was 5′-GGCATTGACTGTGCAACTCCT-3′.

After transfection, the cells were cultured for 24 h, and the expression of TM4SF1-AS1 in each group was detected by qRT-PCR to verify the transfection efficiency.

#### MTT assay was applied to determine cell proliferation

Routine collection of MKN28 and SGC7901 cells in the logarithmic growth phase were inoculated into 96-well plates (with a cell density of 1 × 10^3^ cells/well). The cells were grouped into the shTM4SF1-AS1 and shControl groups. After shRNA silencing, six parallel wells were set up in each group and analysed 24, 48 and 72 h after culture. A 10-μL MTT solution was added to each well during detection. After 4 h of continuous culture, the optical density (OD) value was detected at 490 nm by multifunctional enzyme labelling, and the proliferation curves of MKN28 and SGC7901 cells were visualized. The experiment was repeated three times.

#### Transwell invasion experiment

Matrigel invasion chambers stored at − 20 °C were unpacked, removed, transferred to a 24-well plate and equilibrated to room temperature. Serum-free RPMI-1640 medium was used to dilute cells to 2 × 10^5^ cells/mL. Next, 500 μL RPMI-1640 medium (500 μL) containing 10% foetal bovine serum was added into the upper chamber, and 750 μL was added into the lower well. After culture in a cell incubator for 24 h, the cells were removed. After staining with crystal violet, five visual fields were randomly selected under a microscope for quantification.

#### Flow cytometry was applied to detect apoptosis

MKN28 cells with a confluency rate of approximately 80% in the logarithmic phase were inoculated into 6-well plates (with a density of 4 × 10^5^ cells/well). At the same time, the cells were grouped into shTM4SF1-AS1 and shControl groups, with 3 parallel wells in each group. After 48 h of culture, the cells were collected for apoptosis detection. Two groups of cells were made into single cell suspensions and washed with PBS 3 times. Then, Annexin V-FITC (5 μL) and PI (10 μL) were added and reacted at room temperature in the dark for 5 min. The apoptosis rate was determined using BD flow cytometry (the experiment was completed within 30 min), and the number and ratio of apoptotic cells were calculated. The experiment was repeated three times.

#### Western blot

Cells in each group were lysed, and the protein concentration was determined using the BCA method. SDS-PAGE gels were prepared, proteins were transferred to PVDF membranes and 5% skimmed milk powder was added to block for 3.5 h. Corresponding primary antibodies E-cadherin (1:200), N-cadherin (1:200), Snail (1:200), Twist1 (1:200), β-actin (1:800), TM4SF1 (1:200), p-PI3K (1:300) and p-AKT (1:300) were incubated overnight and washed with PBS 3 times for 10 min each time. Then, HRP anti-rabbit IgG (1:500) was applied and incubated for 1 h at room temperature. ECL chemiluminescence solution was applied for colour development. Grey values were analysed using ImageJ V1.49.

#### Statistical methods

SPSS18.0 was applied for analysis. The data are expressed as the mean ± standard deviation. A *t*-test was used for the comparison of mean values between two groups. Variance analysis was used for the comparison of mean values among multiple groups. The difference was statistically significant when *P* < 0.05.

## Results

### Comparison of LncTM4SF1-AS1 in GC tissues and cell lines

**Fig. 1:**
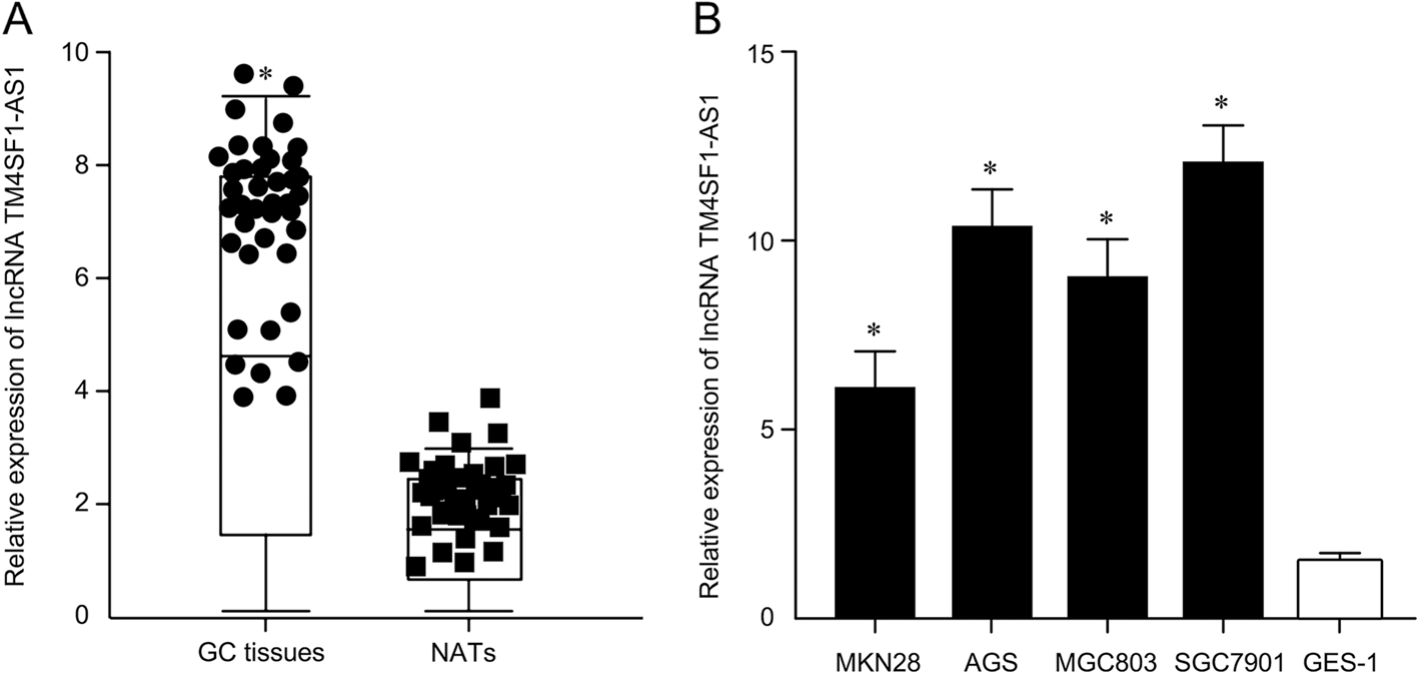
Comparison of the expression levels of lncTM4SF1-AS1 in GC tissues and cell lines. **A** Comparison of lncTM4SF1-AS1 in GC tissues and adjacent tissues. GC tissues, gastric cancer tissues; NATS, normal adjacent tissues; *P < 0.05. **B** LncTM4SF1-AS1 in MKN28, AGS, MGC803, SGC7901 and GES-1

The lncTM4SF1-AS1 level in GC tissues was significantly higher than that in adjacent tissues (7.08 ± 1.09 vs. 2.01 ± 0.24, *P* < 0.05). qRT-PCR experiments revealed that LncTM4SF1-AS1 expression in the GC cell lines MKN28, AGS, MGC803 and SGC7901 was significantly higher than that in the normal gastric mucosa cell line GES-1 (*F* = 24.03, *P* < 0.05). In the four GC cell lines, upregulation of lncTM4SF1-AS1 in MKN28 and SGC7901 cell lines was the lowest and the highest, respectively, so MKN28 and SGC7901 were selected for subsequent cell transfection experiments, as shown in Fig. 1.

### Effect of downregulation of LncTM4SF1-AS1 on the proliferation of GC cells

**Fig. 2:**
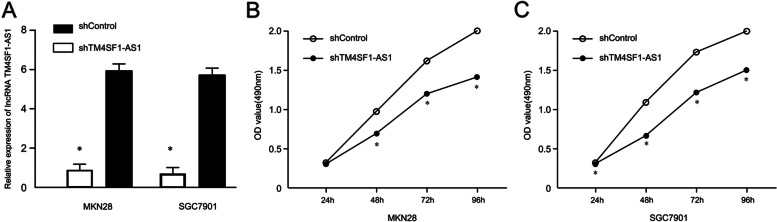
Effects of silencing lncTM4SF1-AS1 on the proliferation of the GC cell lines MKN28 and SGC7901. **A** Measurement of silencing efficiency. **B** MTT assay showing cell proliferation curves of the shTM4SF1-AS1 group and shControl group. *P < 0.05

In the MKN28 and SGC7901 GC cell lines, after shRNA silencing of lncTM4SF1-AS1, expression levels of lncTM4SF1-AS1 in the shTM4SF1-AS1 group were lower than those in the shControl group (*P* < 0.05), as shown in Fig. 2A. This result indicates that the silencing was successful and that the follow-up test could continue. MTT proliferation assay showed that OD values at 490 nm of the shTM4SF1-AS1 group were significantly lower than that of the shControl group at 48 h, 72 h and 96 h, suggesting that downregulation of lncTM4SF1-AS1 inhibited the proliferation of GC cells, as shown in Fig. 2B.

### Effect of downregulation of lncTM4SF1-AS1 on the invasion ability of GC cells

**Fig. 3:**
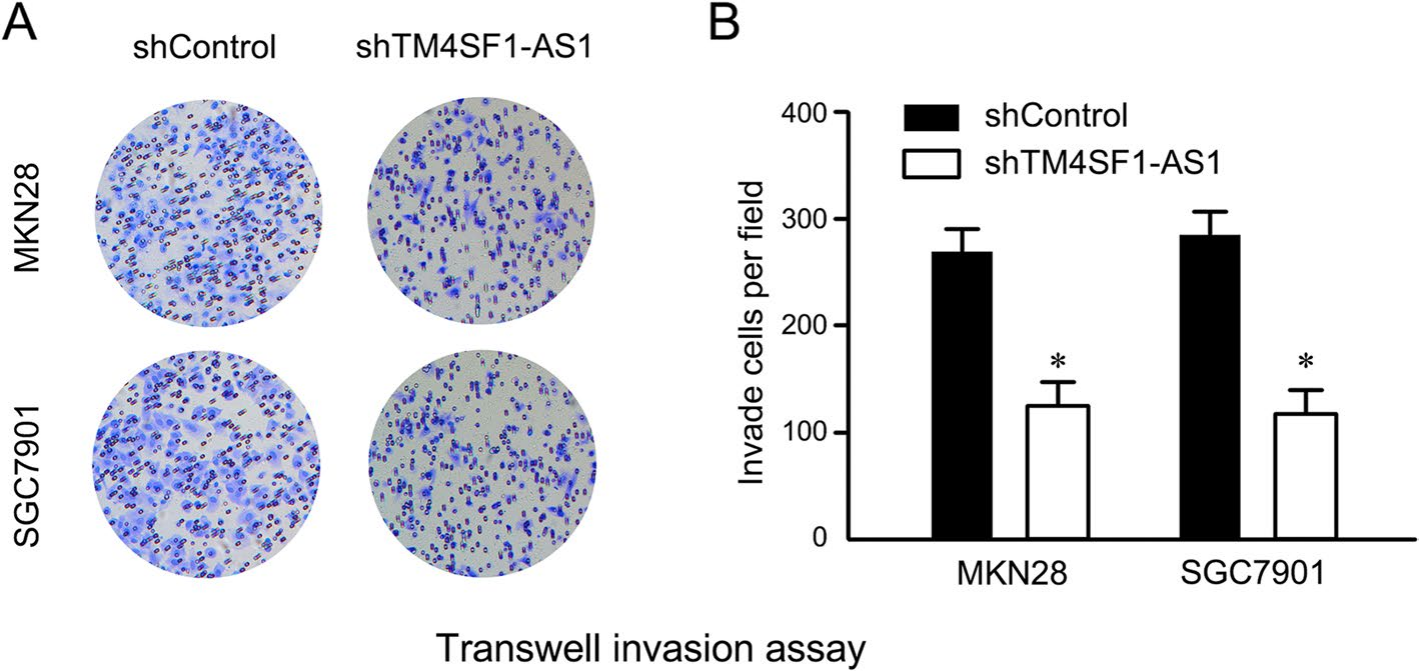
Effect of silencing lncTM4SF1-AS1 on the invasion of GC cell lines MKN28 and SGC7901. **A** Transwell experiments showing that downregulation of lncTM4SF1-AS1 affected invasion. **B** Comparison of the number of invaded cells between the shTM4SF1-AS1 and shControl groups. *P < 0.05

Transwell experiments revealed that after downregulation of lncTM4SF1-AS1, the number of invading cells in the shTM4SF1-AS1 group was significantly less than that in the shControl group (*P* < 0.05), as shown in Fig. 3.

### Effect of downregulation of lncTM4SF1-AS1 on the apoptosis of GC cells

**Fig. 4:**
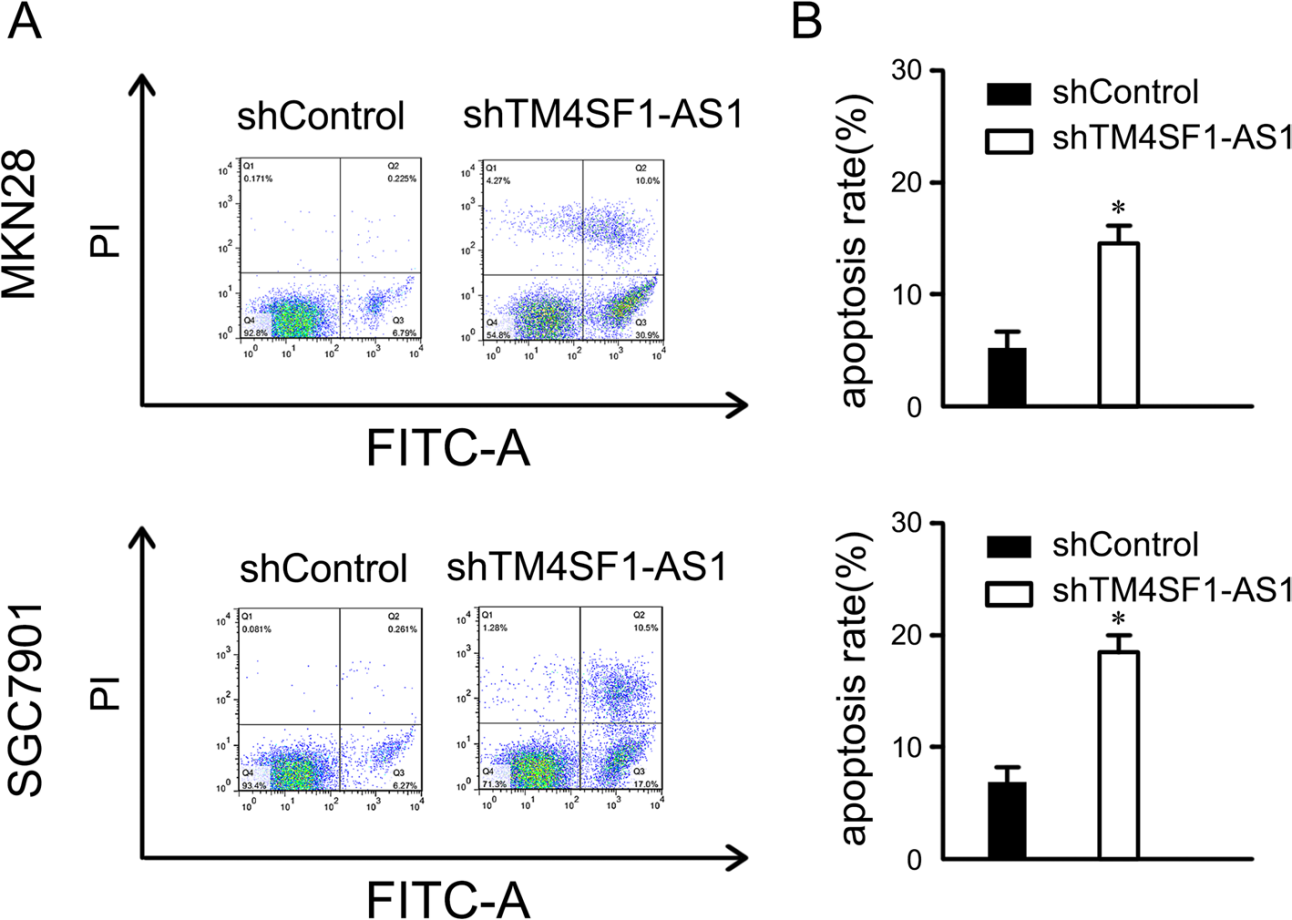
Effect of silencing lncTM4SF1-AS1 on the apoptosis of GC cells. **A** Flow cytometry. **B** Comparison of the apoptosis rate between the shTM4SF1-AS1 and the shControl groups. *P < 0.05

Flow cytometry showed that the apoptosis rate of MKN28 and SGC7901 cells increased after downregulation of lncTM4SF1-AS1, which was significantly higher than that of the shControl group (*P* < 0.05). This result suggests that downregulation of lncTM4SF1-AS1 promotes the apoptosis of MKN28 and SGC7901 cells, as shown in Fig. 4.

### Effect of downregulation of lncTM4SF1-AS1 on the expression of EMT markers in GC

**Fig. 5:**
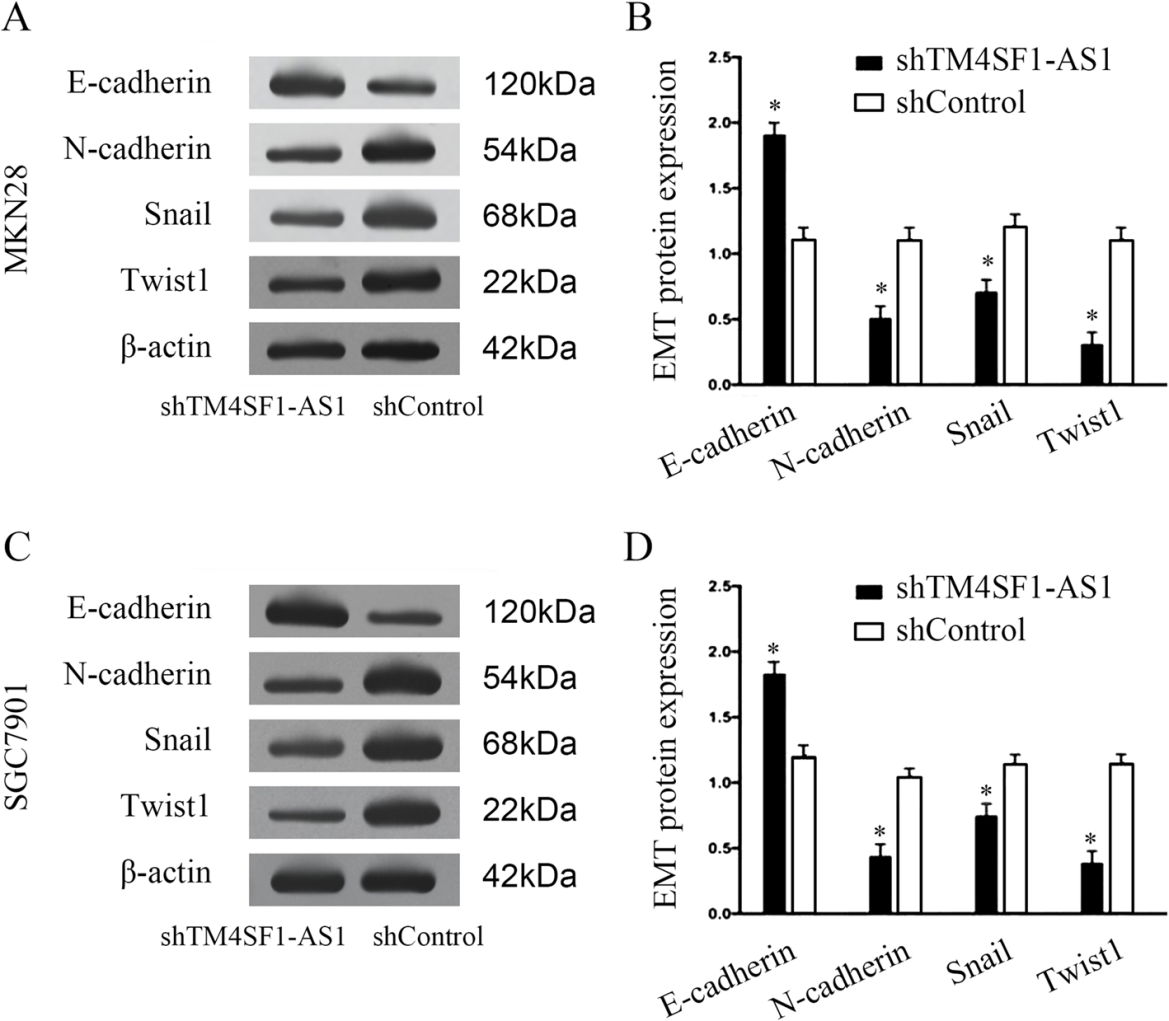
Effect of silencing lncTM4SF1-AS1 on the expression of EMT molecular markers in GC cells. **A** Western blot showing expression levels of EMT molecular markers in MKN28 and SGC7901 cell lines. **B** Comparison of expression levels of EMT molecular markers between the shTM4SF1-AS1 and shControl groups. *P < 0.05

Western blot analysis showed that after downregulating lncTM4SF1-AS1, the expression of EMT metastasis-related proteins in MKN28 and SGC7901 cell lines, which represents the level of E-cadherin in epithelial cells, was significantly higher than that in the shControl group (*P* < 0.05). Levels of the key proteins N-cadherin, Snail and Twist1, which represent interstitial markers, were significantly downregulated (*P* < 0.05). The results showed that downregulation of lncTM4SF1-AS1 inhibited the EMT process in GC cells, as shown in Fig. 5.

### The effect of downregulation of lncTM4SF1-AS1 expression on the PI3K/AKT signalling pathway

**Fig. 6:**
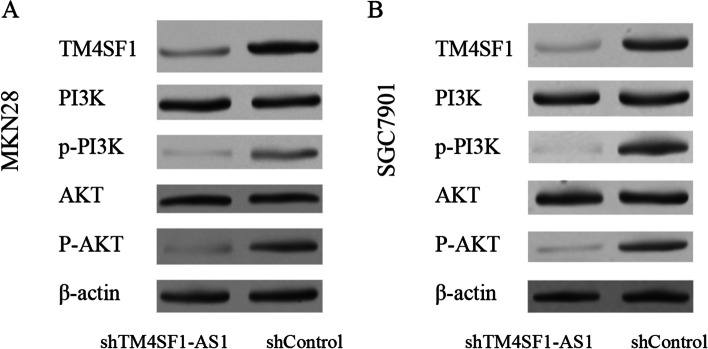
The effect of downregulating the expression of lncTM4SF1-AS1 on the PI3K/AKT signalling pathway. **A** MKN28 cell line. **B** SGC7901 cell line

LncTM4SF1-AS1 is the antisense RNA of TM4SF1. TM4SF1 is a small plasma membrane glycoprotein that belongs to the transmembrane 4 superfamily. TM4SF1 was identified as an oncogene protein and was shown to be correlated with tumour cell metastasis [11, 12]. The literature has reported that after silencing TM4SF1, the expression of the PI3K/AKT signalling pathway was significantly decreased [13]. This study found that after silencing the expression of lncTM4SF1-AS1, the expression of TM4SF1 protein was significantly downregulated, and the expression of p-PI3K and p-AKT signalling proteins was significantly downregulated. There was no significant difference in the expression of PI3K or AKT proteins, as shown in Fig. 6. Silencing LncTM4SF1-AS1 inhibited the PI3K/AKT signalling pathway, and the PI3K/AKT signalling pathway was closely related to invasion, metastasis and the EMT. This suggests that silencing lncTM4SF1-AS1 may lead to downregulated TM4SF1, inhibiting the PI3K/AKT signalling pathway and inhibiting gastric cancer cell invasion, metastasis and the EMT (Fig. 6).

## Discussion

As the fourth most common carcinoma in the world, GC causes 700,000 deaths every year [14]. Chemotherapy has a poor curative effect on unresectable or metastatic GC, resulting in a 5-year survival rate of less than 20% in advanced GC. Therefore, it is urgent to identify new biomarkers and therapeutic targets to improve the early diagnosis and prognostic evaluation of GC [15]. Long noncoding RNA (lncRNA) is a key regulator involved in cell growth, proliferation and apoptosis and even the occurrence and progression of diseases (including carcinomas) [16]. lncRNAs can act as signal mediators, molecular bait, scaffolds or transcription enhancers and participate in the process of carcinoma occurrence and progression [17]. An in-depth study of lncRNA function revealed that it is particularly important to identify molecular markers and therapeutic targets for the diagnosis and prognosis of GC [18].

TM4SF1-AS1 is a 754-bp lncRNA that was first identified and found in the screening of lncRNAs related to the metastasis of NSCLC [19]. An in vitro study of NSCLC revealed that it is highly expressed in non-small cell lung cancer lines and promotes invasion and metastasis of carcinoma cells [9]. In high-throughput RNA sequencing, it was also found that lncTM4SF1-AS1 was significantly increased in colorectal carcinoma regulated and induced by hypoxia [20], and its function is still under exploration. As a newly identified lncRNA, lncTM4SF1-AS1 in GC and its effects on the proliferation, apoptosis and EMT process of GC cell lines in vitro were explored.

The expression of lncTM4SF1-AS1 was compared between carcinoma tissues and adjacent tissues. It was found that lncTM4SF1-AS1 in carcinoma tissues was significantly higher than that in adjacent tissues. LncTM4SF1-AS1 in four GC cell lines, MKN28, AGS, MGC803 and SGC7901, was also significantly higher than that in normal gastric mucosa cells. The above results suggest that lncTM4SF1-AS1 was upregulated in GC, which is similar to that reported in the literature in NSCLC [9] and breast carcinoma [10]. In view of its upregulation in GC tissues and cells, this study selectively downregulated lncTM4SF1-AS1 in GC cell lines. The proliferation of the GC cell line MKN28 was significantly downregulated after the downregulation of lncTM4SF1-AS1, suggesting that downregulation and silencing of lncTM4SF1-AS1 reduces the proliferation of GC cells.

In this research, the effect of lncTM4SF1-AS1 on the invasion of GC cells was investigated. The number of invasive GC cells was significantly decreased after downregulating and silencing lncTM4SF1-AS1 compared to the shControl group. This indicated that lncTM4SF1-AS1 participates in the invasion process of GC and may play a role as an oncogene in GC. The apoptosis of GC cells was significantly higher than that of the control group after downregulating and silencing lncTM4SF1-AS1, suggesting that high expression of lncTM4SF1-AS1 inhibits the apoptosis of GC cells. The occurrence and progression of carcinoma are closely related to the proliferation and apoptosis of carcinoma cells [21], suggesting that targeted inhibition of lncTM4SF1-AS1 promotes the apoptosis of GC cells, which may be a potential therapeutic target for GC.

The epithelial-mesothelial transition (EMT) refers to a process in which the characteristics of epithelial cells gradually disappear and begin to show the characteristics of interstitial cells. Studies have shown that the EMT is closely related to the high invasion and metastasis of carcinoma cells [22, 23]. An increasing amount of evidence has revealed that lncRNAs participate in the occurrence and progression of carcinoma by regulating the EMT of carcinoma cells, play the role of oncogene promoters or tumour suppressors and become key regulators of the migration, invasion and metastasis of tumour cells [24]. After downregulating and silencing lncTM4SF1-AS1, the expression levels of the E-cadherin protein, a marker protein of epithelial properties, were significantly increased, while the levels of N-cadherin, Snail and Twist 1 protein related to mesothelial properties were significantly decreased during the EMT. This result suggests that downregulating and silencing lncTM4SF1-AS1 inhibits the invasion of GC cells by changing key proteins in the EMT pathway. Currently, there are few reports on the role of lncTM4SF1-AS1 in tumours. Studies have shown that high lncTM4SF1-AS1 activates the PI3K/AKT signalling pathway in lung carcinoma A549 cells in vitro and promotes the proliferation and invasion of carcinoma cells [9]. Our findings suggest that downregulating and silencing lncTM4SF1-AS1 changes EMT-related proteins and inhibits the proliferation of GC cells, suggesting that it may represent a potential therapeutic target for GC [25].

Many cell signalling pathways regulate the EMT process, including the Notch1 signalling pathway, TGF-β signalling pathway, Wnt signalling pathway and PI3K/AKT signalling pathway. The PI3K/AKT signalling pathway can regulate tumour cell proliferation, apoptosis, differentiation and metastasis [26–29]. Studies have reported that [30, 31] many lncRNA molecules can regulate PI3K/AKT cell signalling pathways, such as lncRNA-HOTAIR and lncRNA FOXO1. The lncTM4SF1-AS1 gene is located on human chromosome 3q25.1 and is the antisense RNA of TM4SF1, while TM4SF1 is an oncogene that promotes tumour cell metastasis [11, 12]. The results of this study found that silencing lncTM4SF1-AS1 downregulates the expression of TM4SF1 protein. It also silences the expression of p-PI3K and p-AKT signalling proteins in the PI3K/AKT signalling pathway, indicating that silencing lncTM4SF1-AS1 can downregulate TM4SF1 and silence the PI3K/AKT signalling pathway. This also explains the inhibitory effects of silencing lncTM4SF1-AS1 on the proliferation, metastasis and EMT of gastric cancer cells.

However, this study also has limitations. A functional study of lncTM4SF1-AS1 in gastric cancer animal models was not performed, which is our next research direction.

## Conclusions

In summary, lncTM4SF1-AS1 is upregulated in GC tissues and carcinoma cell lines. Downregulation of lncTM4SF1-AS1 inhibits the proliferation and promotes the apoptosis of GC cells and can participate in the EMT process, which is a latent target for GC. Further study on the molecular mechanism of lncTM4SF1-AS1 involved in the occurrence and development of GC will be helpful for the early clinical diagnosis of GC and the development of targeted drugs.

## Data Availability

The datasets used and/or analysed during the current study are available from the corresponding author on reasonable request.
